# Birth trauma in preterm spontaneous vaginal and cesarean section deliveries: A 10-years retrospective study

**DOI:** 10.1371/journal.pone.0275726

**Published:** 2022-10-17

**Authors:** Alina Luca, Angela Vinturache, Ciprian Ilea, Andreea Avasiloaiei, Luminita Paduraru, Alexandru Carauleanu, Ioana Sripcariu, Demetra Socolov

**Affiliations:** 1 Department of Obstetrics & Gynecology, Gr. T. Popa University of Medicine and Pharmacy, Iasi, Romania; 2 CuzaVoda Maternity University Hospital, Iasi, Romania; 3 Department of Obstetrics and Gynecology, University of Alberta & Department of Nuroscience, University of Lethbridge, Alberta, Canada; 4 Department of Obstetrics & Gynecology, Grande Prairie Regional Hospital, Alberta, Canada; National Institute of Perinatology: Instituto Nacional de Perinatologia, MEXICO

## Abstract

**Objective:**

We compared birth injuries for spontaneous vaginal (VD) and caesarean section (CS) deliveries in preterm and term pregnancies.

**Methods:**

A retrospective cohort study was conducted in a single tertiary center, between January 1st, 2007, and December 31st, 2017. The study included 62330 singleton pregnancies delivered after 24 0/7 weeks gestation. Multivariable analyses compared trauma at birth, birth hypoxia and birth asphyxia in term and preterm deliveries, stratified by mode of birth, VD versus CS. Main outcome measure was trauma at birth including intracranial laceration and haemorrhage, injuries to scalp, injuries to central and peripheral nervous system, fractures to skeleton, facial and eye injury.

**Results:**

The incidence of preterm deliveries was 10.9%. Delivery of preterm babies by CS increased from 37.0% in 2007 to 60.0% in 2017. The overall incidence of all birth trauma was 16.2%. When stratified by mode of delivery, birth trauma was recorded in 23.4% of spontaneous vaginal deliveries and 7.5% of CS deliveries (aOR 3.3, 95%CI 3.1–3.5). When considered all types of birth trauma, incidence of trauma at birth was higher after 28 weeks gestation in VD compared to CS (28–31 weeks, aOR 1.7, 95% CI 1.3–2.3; 32–36 weeks, aOR 4.2, 95% CI 3.6–4.9; >37 weeks, aOR 3.3, 95% CI 3.1–3.5). There was no difference in the incidence of birth trauma before 28 weeks gestation between VD and CS (aOR 0.8, 95% CI 0.5–1.2). Regarding overall life-threatening birth trauma or injuries at birth with severe consequences such as cerebral and intraventricular haemorrhage, cranial and brachial nerve injury, fractures of long bones and clavicle, eye and facial injury, there was no difference in vaginal preterm deliveries compared to CS deliveries (p > 0.05 for all).

**Conclusion:**

CS is not protective of injury at birth. When all types of birth trauma are considered, these are more common in spontaneous VD, thus favoring CS as preferred method of delivery to avoid trauma at birth. However, when stratified by severity of birth trauma, preterm babies delivered vaginally are not at higher risk of major birth trauma than those delivered by CS.

## Introduction

Birth trauma is defined as all mechanical or hypoxic neonatal injuries sustained during labour and delivery. The rate of birth trauma was estimated anywhere between 2.45 and 41.16 per 1000 births. The functional sequelae remain responsible for a significant neonatal morbidity and mortality [1, 2].

Infant mortality result of birth trauma has declined over the past decades in comparison with historical references, reflecting in part the technological advancements in obstetrics that allow early recognition of risk factors using ultrasonography and fetal monitoring [3]. Most of the evidence to date demonstrates a lower rate of birth trauma after caesarean section (CS) compared to spontaneous vaginal delivery (VD). The relative benefit of CS will be stronger in neonates born late preterm [4]. While the data is more homogeneous for term births, there is still controversy on the preferred mode of delivery (VD or CS) for preterm babies, in relation to fetal and neonatal death and trauma at birth with arguments on the both sides [5–10].

This study was designed to assess the contemporaneous frequency of birth trauma and its consequences in singleton preterm births, in cephalic presentation. We hypothesized that delivery of preterm fetuses by CS would be associated with a lower incidence of neonatal birth trauma. Thus, we examined the rates of birth trauma, using all codes of *International Classification of Diseases*, *Tenth Revision* (ICD-10) from the birth trauma section, codes that represent outcomes considered avoidable through practice modification. It is our hope that the findings current study will contribute to advance the knowledge in the field, facilitating changes in medical practice and offering support in the decision-making process for obstetricians regarding the mode of delivery for preterm babies.

## Materials and methods

### Data source

We performed a 10-year retrospective cohort study of all singleton spontaneous cephalic deliveries from a single tertiary center in Romania, between January 1st, 2007 and December 31st, 2017 on. Our university maternity hospital serves as referral center for all maternity hospitals in northeast Romania. We used as data source the electronic medical records of hospital admissions for delivery from 67,348 mother and baby dyads.

Information of maternal sociodemographic characteristics, obstetrical events at delivery, and neonatal outcomes was abstracted from the electronic medical records. The information retrieved was based on hospital discharge diagnosis codes, classified using the International Statistical Classification of Diseases and Related Health Problems, version 2010 (ICD-10). Additional details about the ICD-10 codes are provided in the Appendix (S1 and S2 Tables). The data was anonymized before distribution to the study team.

Detailed data related to demographics (mother’s age, place of origin, parity, ethnicity, maternal comorbidities), pregnancy (gestational age, fetal presentation, mode of birth, days of hospitalization), and newborn (birth trauma due to birth injury, hypoxia, asphyxia, Apgar score, and days of admission in neonatal care unit (NICU) were abstracted from the electronic database.

### The inclusion criteria

Our study population included women with singleton, cephalic presentation, who delivered preterm, between 24 + 0/7 weeks to 36 + 6/7 weeks gestation, and at term, ≥ 37+0/7 weeks gestation. Women were excluded from this study if they had multiple pregnancies, presentation other than cephalic, newborns who were not delivered in our center, newborns with pre-existing condition or congenital malformations. Women with missing information were excluded from the study. Infants delivered by vacuum or forceps were excluded from this analysis due to the known possibility that the operative deliveries could independently increase morbidity and mortality for those neonates. Only women who had a CS or spontaneous VD were included in the study.

The final study population was represented by 62.330 mother-newborn dyads.

### Variables

#### Pregnant women and newborns grouping

Using World Health Organization classification of preterm birth, the participants were classified in four groups based on the gestational age at birth: extremely preterm, 24 + 0/7 to 27+ 6/7 weeks; very preterm, 28 + 0/7 to 31+ 6/7 weeks; moderate to late preterm 32 + 0/7–36 + 6/7 weeks, and term pregnancies, 37 + 0/7–42 + 0/7 weeks. Routine first trimester dating US was used to calculate estimated date of delivery and gestational age at delivery.

Maternal sociodemographic characteristics were categorized as follows: maternal age was divided in five categories (less than 20, 21–24, 25–30, 31–34, and ≥35 years old); ethnicity in two categories (white non-Hispanic, other), parity in two categories (nulliparous, multiparous), location in two categories (urban, rural). Maternal comorbidities included in the full model as confounding variables included pre-existing diabetes mellitus, gestational diabetes mellitus, chronic hypertension, pregnancy-induced hypertensive disorders, preterm labour, preterm rupture of membranes, chorioamnionitis, and urinary tract infection. We combined diabetes and gestational diabetes for the simplicity of the analysis. Pregnancy-induced hypertensive disorders included pregnancy induced hypertension, preeclampsia, and HELLP syndrome. There was a small number of women with pre-existing hypertension, and this group was amalgamated with pregnancy-induced hypertensive disorders for meaningful comparisons.

The mode of birth was classified in two categories: CS and VD, with CS delivery used as the reference category.

The year of delivery was included in the descriptive analyses to illustrate how the incidence of CS delivery changed over time in our cohort.

#### Neonatal birth trauma classification

Cerebral hemorrhage due to birth trauma, included subdural, cerebral, subarachnoid, tentorial hemorrhage, lacerations, and intracranial hemorrhage.

Intraventricular hemorrhage included grades I, II, and III hemorrhage.

“Other injury to scalp” category of birth trauma included cephalohematoma, caput succedaneum, epicranial hemorrhage, and lesions of the scalp.

Several other types of traumata were analyzed, including skull fracture, fracture of clavicle, long bones, injury to brachial plexus, hematoma or rupture of the spleen, hematoma or rupture of the liver, injury, or damage to the eye(s), facial injury, fetal laceration by scalpel, ecchymosis, and subcutaneous adiponecrosis.

Other birth injuries analyzed as individual outcomes included hypoxia and asphyxia at birth; intrapartum and peripartum fetal death.

Apgar scores and 5 and 10 minutes were divided in two categories: ≤7 and > 7.

The outcome variables that assessed fetal birth trauma were defined as follows: hypoxia, O_2_ concentration in umbilical cord <22mEq/L; birth asphyxia, arterial pH <7.0; APGAR score less than 3 at 5 and 10 min, signs of hypoxic ischemic encephalopathy, evidence of multi-organ suffering.

#### Outcomes measures

The main outcome of this study was trauma at birth which included intracranial laceration and haemorrhage, injuries to scalp, injuries to central and peripheral nervous system, fractures to skeleton, and facial and eye injury.

The secondary outcomes included intrauterine hypoxia, birth asphyxia, neonatal aspiration syndromes and neonatal death, subcutaneous adiponecrosis, ecchymosis, fetal laceration by scalpel. ICD-10 codes used for specific diagnoses are listed in Appendix (S*upplementary material A*). In order to better assess the overall impact of mode of birth on the newborn outcomes in term and preterm deliveries, we merged all types of birth trauma and created a new variable, “all birth trauma”. Mother and newborn days of hospitalisation, and the risk of admission to neonatal intensive care unit (NICU) were also assessed.

The outcomes were compared between the CS and VD and stratified by gestational age.

### Statistical analysis

Descriptive statistics were produced for all study variables. Continuous variables were presented as mean (95% confidence interval) ± standard deviation (SD). Categorical variables were presented as proportions.

Bivariate analysis was performed to examine the associations between the mode of birth, demographic characteristics and clinical outcomes in term and preterm pregnancies. Chi square test was used for categorical variables and ANOVA for continuous variables, as appropriate.

Multivariate logistic regressions assessed the association between mode of delivery and birth trauma in term and preterm pregnancies adjusting for potential confounding variables. Term pregnancy and delivery by CS were the reference categories. Confounding factors that remained in the multivariable models and were adjusted for included maternal demographic and clinical characteristics (age, location, ethnicity, parity, gestational age) maternal pre-existing pathology (diabetes, hypertension, genital tract infection), and pregnancy complications (hypertensive disorders, gestational diabetes, premature rupture of membranes, chorioamnionitis).

Statistical analysis was conducted using SPSS statistical software, version 20 for Windows. Where appropriate unadjusted (OR) and adjusted odds ratio (aOR) and their 95% confidence intervals (CI) were computed; a nominal P-value of less than 0.05 was considered statistically significant.

### Ethics statement

The study was approved by the ethical committee of “Cuza-Voda” University Hospital, No 2052/16.02.2021 and “Gr T Popa” University of Medicine and Pharmacy, No 101/08.07.2021 Iasi, Romania, and was performed in line with the Declaration of Helsinki.

Signed informed consent from patients was not required for this retrospective study that used a denominated data set for secondary data analysis. All patients admitted to hospital provided informed written consent to have data from their anonymized medical records used in research as pooled data.

## Results

From 67,348 women who delivered at our institution between January 2007 and December 2017, 62,330 women met the criteria of inclusion in the study. From these, 6,794 (10.9%) were preterm deliveries. Among women included in the study, 28,191(45.2%) were delivered by CS and 34,139 (54.8%) had a spontaneous vaginal delivery. Approximately 63% of women who delivered by CS were primiparous. Baseline demographic and clinical characteristics of the study population are presented in Table 1. The majority of women who delivered by CS were 30 years and older, whereas women 20 years and younger were more likely to have a VD. Women from urban area were three times more likely to deliver by CS compared to women from rural communities (OR 3.1; 95% CI 2.9–3.2).

**Table 1:** Demographic and clinical characteristics of the study population, stratified by delivery mode and gestational age at delivery.

	Cesarean Delivery* n = 28,191	Vaginal Delivery n = 34,139	OR (95% CI)	p-value	Preterm births n = 6794	Term births n = 55415	OR (95% CI)	p-value
**Maternal age (mean, SD)**	**29.3 (5.4)**	**26.7 (5.9)**	0.90(0.92-.93)	<0.0001	**28.6 (6.3)**	**27.8 (5.8)**	0.9 (0.97–0.98)	<0.0001
under 20	1.904 (6.8%)	5.856 (17.2%)	4.2 (3.9–4.5)	<0.0001	854 (12.6%)	6.888(12.4%)	1.4 (1.3–1.6)	<0.0001
21–24 ys old	3.185 (11.3%)	6.761 (19.8%)	2.9 (2.7–3.1)	<0.0001	904 (13.3%)	9.019 (16.3%)	1.8(1.6–1.9)	<0.0001
25–30 ys old	11.421 (40.5%)	12.517 (36.7%)	1.5 (1.4–1.6)	<0.0001	2.315 (34.1%)	21.584 (38.9%)	1.7 (1.5–1.8)	<0.0001
31–34 ys old	6.828 (24.2%)	5.485 (16.1%)	1.1 (1.04–1.2)	<0.0001	1.434 (21.1%)	10.850 (19.6%)	1.3(1.2–1.5)	<0.0001
over 35	4.853 (17.2%)	3.520 (10.3%)	1*	<0.0001	1.287 (18.9%)	7.074 (12.8%)	1*	<0.0001
**Parity (n, %)**								
Primiparous	17.807 (63.2%)	20.809 (60.9%)	1*		3.474 (51,1%)	34.975 (63,1%)	1*	
Multiparous	10.384 (36.8%)	13.330 (39.1%)	1.06 (0.97–1.08)	0.045***	3.320 (48,9%)	20.440 (36,9%)	0.7 (0.6–0.8)	<0.0001
**Location n (%)**								
Urban	18.293 (64.9%)	12.812 (37.5%)	1*		3.415 (50.3%)	27.633 (49.9%)	1*	
Rural	9.898 (35.1%)	21.327 (62.5%)	3.0 (2.9–3.2)	<0.0001	3.379 (49.7%)	27.782 (50.1%)	1.01 (0.9–1.07)	0.530
**Gestational age at delivery (weeks)^a^ (n, %)**								
24–27 weeks	57 (0.2%)	145 (0.4%)	2.0 (1.5–2.8)	<0.0001	202 (3.0%)	-	-	-
28–31 weeks	298 (1.1%)	434 (1.3%)	1.1 (1.01–1.4)	0.037***	732 (11.0%)	-	-	-
32–36 weeks	3.028 (10.8%)	2.679 (7.9%)	0.7 (0.6–0.8)	<0.0001	5.707 (85.9%)	-	-	-
≥ 37 weeks	24.701(88.0%)	30.714 (90.4%)	1*	-	-	55.415 (100%)	-	-
**Maternal comorbidities (n, %)**								
Diabetes^b^	537 (1.9%)	143 (0.4%)	0.2 (0.1–0.4)	<0.0001	145 (2.1%)	531 (1.0%)	0.4 (0.3–0.5)	<0.0001
Hipertensive disorders^c^	1.139 (4.0%)	731 (2.1%)	0.5 (0.4–0.6)	<0.0001	378 (5.6%)	1.490 (2.7%)	0.5 (0.4–0.5)	<0.0001
Premature rupture of membranes	4.296 (15.2%)	6.785 (19.9%)	1.38(1.32–1.4)	<0.0001	2.358 (34.7%)	8.702 (15.7%)	0.35 (0.33–0.37)	<0.0001
Chorioamnionitis	484 (1.7%)	866 (2.5%)	1.5 (1.3–1.7)	<0.0001	475 (7.0%)	874 (1.6%)	0.2 (0.19–0.24)	<0.0001
Genital tract infection	2.076 (7.4%)	1.924 (5.6%)	0.75 (0.7–0.8)	<0.0001	481 (7.1%)	3.510 (6.3%)	0.89(0.80–0.98)	0.018***

Abbreviations: OR unadjusted odd s ratio.

Data presented as n (%) for categorical variables or mean (95% confidence intervals)

*Reference category

**p<0.001

***p<0.05.

^a^Sample size does not equal to 62.330 due to missing data

^b^includes pre-existing diabetes mellitus and gestational diabetes mellitus.

^c^include hypertension with onset during pregnancy, pre-existing hypertension, and preeclampsia.

**Fig 1:**
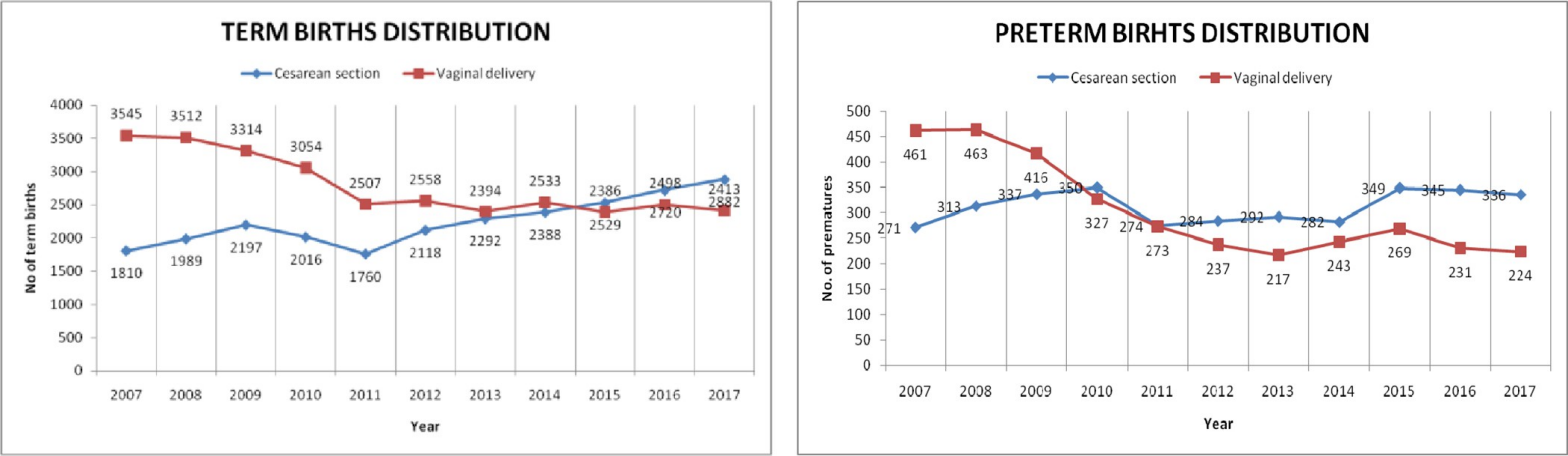
Distribution of preterm and term births delivered by CS vs VD, in one tertiary center, from January 1st, 2007, and December 31st, 2017.

As shown in Fig 1, until 2011 more women delivered preterm vaginally. While at the beginning of the study interval, 62.9% of preterm deliveries were vaginal deliveries, the ratio in 2017 was 1.5 to 1.0 in favor of CS. Women who delivered preterm were overall older than women who had term deliveries, with an average age of 28.6 years compared to 27.8 years of term pregnancies. The percentage of preterm deliveries was higher in multiparous women. One third of preterm deliveries were associated with PPROM. For term deliveries, the trend of changes in mode of birth was similar: 66.2% of term deliveries in 2007 were vaginal deliveries, while by 2017 the ratio turned to 1.19 to 1.0 in favor of CS. Delivery by CS was more common among women with diabetes (OR 0.2, 95% CI 0.1–0.4; p<0.0001) and hypertensive disorders (OR 0.5, 95% CI 0.4–0.7, p<0.0001), whereas women with PROM and chorioamnionitis were more likely to have a VD. When stratified by GA at delivery, CS was preponderant in preterm deliveries in all the above complications (p<0.0001 for all).

Table 2 shows the clinical outcomes in women with CS and VD stratified by gestational age at birth.

**Table 2:** Maternal and neonatal birth outcomes for preterm and term, caesarean section and spontaneous vaginal deliveries stratified by gestational age.

Variable	Gestational age at delivery (weeks)	CS* (n = 28,191)	VD (n = 34,139)	OR (95% CI)	p-value	aOR (95% CI)	p-value
**Duration of hospitalization for mothers (days)^1^** (no of days, %)	24+0 to 27+6	12.9 (57; 8.8)	10.5 (145, 9.1)	-	-	-	-
	28+0 to 31+6	13.3 (298; 9.2)	11.2 (434, 9.4)	-	-	-	-
	32+0 to 36+6	10.8 (3,028; 10.1)	7.8 (2,679, 5.7)	-	-	-	-
	all preterms	11.0 (3,433; 9.9)	8.3 (3,361, 6.6)	-	-	-	-
	≥37+0	5.9 (24,701; 5.1)	4.9 (30,714, 3.5)	-	-	-	-
**Duration of hospitalization for newborns^1^ (no of days, %)**	24+0 to 27+6	61.5 (57; 41.4)	65.0 (145, 39.0)	-	-	-	-
	28+0 to 31+6	51.7 (298; 24.0)	47.1 (434, 22.2)	-	-	-	-
	32+0 to 36+6	12.3 (3,028; 11.8)	12.3 (2,679, 11.5)	-	-	-	-
	all preterms	16.5 (3,433; 18.9)	18.8 (3,361, 21.6)	-	-	-	-
	≥37+0	4.3 (24,701; 2.5)	3.9 (30,714, 2.4)	-	-	-	-
**NICU^2^**	24+0 to 27+6*	9 (15.8%)	15 (10.3%)	0.6 (0.3–1.5)	0.280	0.6 (0.2–1.7)	0.400
	28+0 to 31+6	46 (15.4%)	34 (7.8%)	0.4 (0.3–0.7)	0.001	0.3 (0.2–0.6)	0.0003
	32+0 to 36+6	165 (5.4%)	73 (2.7%)	0.5 (0.4–0.6)	<0.0001	0.4 (0.3–0.6)	<0.0001
	≥37+0	333 (1.3%)	271 (0.9%)	0.6 (0.5–0.8)	<0.0001	0.6 (0.5–0.7)	<0.0001
**Apgar 5’ ≤ 7^a^^2^**	24+0 to 27+6*	57 (2.6%)	145 (3.9%)	**-**	**-**	-	-
	28+0 to 31+6	282 (12.8%)	401 (10.8%)	0.7 (0.4–1.3)	0.280	0.8 (0.4–1.6)	0.610
	32+0 to 36+6	909 (41.1%)	847 (22.8%)	1.1 (0.9–1.2)	0.180	0.9 (0.8–1.1)	0.980
	≥37+0	963 (43.6%)	2.317 (62.5%)	2.0 (1.8–2.2)	<0.0001	1.8 (1.6–1.9)	<0.0001
	Total	2.211 (7.9%)	3.710 (10.9%)	1.4 (1.3–1.5)	<0.0001	1.3 (1.2–1.4)	<0.0001
**Apgar 5’ >7^a^^2^**	24+0 to 27+6*	-	-	-	-	-	-
	28+0 to 31+6	16 (5.4%)	32 (7.4%)	1.4 (0.7–2.6)	0.280	1.2 (0.6–2.3)	0.6
	32+0 to 36+6	2.1 (70.0%)	1.8 (68.3%)	0.9 (0.8–1.03)	0.180	1.01 (0.8–1.1)	0.9
	≥37+0	23.7 (96.1%)	28.4 (92.5%)	0.5 (0.4–0.5)	<0.0001	0.5 (0.5–0.6)	<0.0001
	Total	25.863 (92.1%)	30.236 (89.1%)	0.7 (0.6–0.7)	<0.0001	0.7 (0.7–0.8)	<0.0001
**Hypoxia**	24+0 to 27+6*	-	-	-	-	-	-
	28+0 to 31+6	-	-	-	-	-	-
	32+0 to 36+6	12 (1.5%)	29 (2.0%)	2.75 (1.4–5.4)	0.003***	2.6 (1.3–5.3)	0.007***
	≥37+0	812 (98.5%)	1.386 (4.5%)	1.39 (1.2–1.5)	<0.0001	1.2 (1.02–1.3)	<0.0001
**Asphyxia^2^**	24+0 to 27+6*	-	-	-	-	-	-
	28+0 to 31+6	2 (9.1%)	1 (4.3%)	0.3 (0.03–3.8)	0.380	0.3 (0.03–3.8)	0.382
	32+0 to 36+6	4 (18.2%)	2 (8.7%)	0.5 (0.1–3.1)	0.510	0.5 (0.09–3.2)	0.502
	≥37+0	16 (72.7%)	20 (87.0%)	1.01 (0.5–1.9)	0.980	0.9 (0.5–1.9)	0.878
**Stillbirth^2^**	24+0 to 27+6	66 (40.7%)	116 (45.0%)	1.7 (1.6–1.7)	<0.0001	-	-
	28+0 to 31+6	32 (19.8%)	55 (21.3%)	1.7 (1.5–1.7)	<0.0001	-	-
	32+0 to 36+6	36 (22.2%)	49 (19.0%)	1.3 (1.2–1.4)	<0.0001	-	-
	≥37+0	28 (17.3%)	38 (14.7%)	1.3 (1.2–1.4)	<0.0001	-	-
	Total	162 (100.0%)	258 (100.0%)	1.6 (1.5–1.7)	<0.0001	-	-
**All traumas^2^**	24+0 to 27+6*	26 (1.2%)	61 (0.8%)	0.8 (0.4–1.6)	0.640	0.9 (0.5–1.8)	0.941
	28+0 to 31+6	73 (3.5%)	144 (1.8%)	1.5 (1.1–2.1)	0.012***	1.4 (1.03–2.1)	0.034***
	32+0 to 36+6	227 (10.8%)	675 (8.4%)	4.2 (3.5–4.8)	<0.0001	4.4 (3.5–4.9)	<0.0001
	≥37+0	1.784 (84.5%)	7.122 (89.0%)	3.8 (3.6–4.1)	<0.0001	3.6 (3.4–3.8)	<0.0001

Abbreviations: OR unadjusted odds ratio, aOR adjusted odds ratio for maternal age, parity, ethnicity, gestational age at delivery, and maternal comorbidities.

^1^Data presented as mean (n, standard deviation).

^2^Data presented as n (%).

*Reference category

**p<0.001

***p<0.05.

^a^Sample size does not equal to 62.330 due to missing date and/or rounding error

Duration of maternal hospitalization was longer in women with CS delivery. There was three days difference for moderate and late preterm deliveries in women delivered by CS compared to VD (10.8 days for CS, 7.8 days for VD) and less, of only one day, in term deliveries (5.9 days CS, 4.9 days VD). In terms of neonatal days of hospitalization, there was no significant differences between VD and CS for both preterm and term babies.

We observed a slightly higher incidence of NICU admission for very preterm deliveries after CS comparing with VD (OR 0.46, 95% CI 0.3–0.7, p = 0.001), as well for moderate to late preterm (OR 0.5, 95% CI 0.3–0.6, p<0.0001). Concerning term deliveries, 1.3% were admitted in NICU after CS vs 0.9% in VD (OR 0.65, 95% CI, 0.5–0.8, p<0.0001). After adjusting for maternal clinical characteristics, CS delivery continued to be associated with increased odds of admission to NICU. In our study, admission in NICU was longer among CS group, except for the extremely preterm babies, were the admission period in NICU was similar between CD and VD (aOR 0.38, 95% CI, 0.2–0.6, p = 0.0003). The median duration of admission in NICU remained increased for both CS and VD in the adjusted model for confounding variables.

For all extreme preterm babies, the Apgar score at 5 min was ≤7. For the preterm births in 28–31 weeks and 32–36 weeks groups, there were no significant differences in Apgar scores at 5 min between VD and CS group. For term pregnancy, Apgar score ≤7 at 5 min was more frequent for VD (6.7% vs 3.4%, p <0.0001), whereas Apgar score >7 at 5 min was more common in CS than VD (84.2% vs 83.1%, p <0.0001); the findings remained similar in the adjusted models.

Fetal hypoxia was more common for VD route of delivery compared to CS (4.1% vs 2.9%, p<0.0001). After adjusting for confounders, the odds of neonatal hypoxia after VD decreased from 39% to 19.7% compared to those delivered by CS (aOR 1.197, 95% CI 1.02–1.3, p<0.0001). There was no significant difference in the risk of developing severe asphyxia between VD and CS delivery. Stillbirth babies were more likely to be delivered vaginally, regardless of the gestational age.

Table 3 shows the results of multivariable analysis assessing the birth trauma in term and preterm deliveries in VD and CS births. When summing up all birth traumatisms, including the minor trauma, there was a total of 10,112 neonatal birth injuries, which represented a rate of 16.2% of all deliveries. The risk of birth trauma increased with increasing gestational age, from 0.86% in the 24–27 weeks age group to 0.88% in the term pregnancies group. At very low gestational age there was no difference in birth trauma incidence between CS and VD deliveries. With increasing gestational age, the trauma at birth became more common in babies who underwent a VD.

**Table 3:** Neonatal trauma stratified by mode of delivery and gestational age at delivery in singleton pregnancies.

Type of trauma	Gestational age at delivery	Cesarean section (n = 28191)	Vaginal delivery (n = 34139)	OR (95% CI)	p-value	aOR (95% CI)	p-value
**Cerebral traumatic hemorrhage**	24+0 to 27+6*	0 (0.0%)	4 (100.0%)	-	0.990	-	0.990
	28+0 to 31+6	1 (50.0%)	1 (50.0%)	0.7 (0.04–11.0)	0.790	0.5 (0.03–7.9)	0.610
	32+0 to 36+6	1 (50.0%)	1 (50.0%)	1.13 (0.07–18.1)	0.930	1.9 (0.1–34.5)	0.650
	≥37+0	0 (0.0%)	0 (0.0%)	-	-	-	-
**Intraventricular haemorrhage**	24+0 to 27+6*	50 (41.7%)	70 (58.3%)	0.7 (0.4–1.4)	0.350	0.7 (0.4–1.5)	0.470
	28+0 to 31+6	101 (50.8%)	98 (49.2%)	0.9 (0.6–1.3)	0.590	0.9 (0.6–1.4)	0.630
	32+0 to 36+6	59 (56.7%)	45 (43.3%)	1.1 (0.7–1.8)	0.500	1.05 (0.6–1.7)	0.820
	≥37+0	7 (58.3%)	5 (41.7%)	0.6 (0.2–1.8)	0.340	0.7 (0.2–2.6)	0.670
**Cranial nerve injury**	24+0 to 27+6*	0 (0.0%)	0 (0.0%)	-	-	-	-
	28+0 to 31+6	0 (0.0%)	0 (0.0%)	-	-	-	-
	32+0 to 36+6	0 (0.0%)	0 (0.0%)	-	-	-	-
	≥37+0	0 (0.0%)	1 (0.0%)	-	-	-	-
**Fracture long bones**	24+0 to 27+6*	0 (0.0%)	0 (0.0%)	-	-	-	-
	28+0 to 31+6	0 (0.0%)	0 (0.0%)	-	-	-	-
	32+0 to 36+6	1 (100.0%)	0 (0.0%)	-	-	-	-
	≥37+0	6 (75.0%)	2 (25.0%)	0.3 (0.06–1.6)	0.130	0.2 (0.03–1.1)	0.070
**Fracture of clavicle**	24+0 to 27+6*	0 (0.0%)	1 (100.0%)	-	-		
	28+0 to 31+6	0 (0.0%)	2 (100.0%)	-	-		
	32+0 to 36+6	3 (25.0%)	9 (75.0%)	4.2 (1.1–15.5)	0.031***	9.2 (1.1–75.4)	0.038***
	≥37+0	21 (5.6%)	352 (94.4%)	14.9 (9.6–23.2)	<0.0001	15.01 (9.3–24.3)	<0.0001
**Other scalp injury**	24+0 to 27+6*	3 (15.8%)	16 (84.2%)	3.3 (0.9–11.6)	0.06	3.9 (0.8–19.8)	0.090
	28+0 to 31+6	15 (15.2%)	84 (84.8%)	6.9 (3.9–12.2)	<0.0001	4.1 (2.2–7.6)	<0.0001
	32+0 to 36+6	190 (22.7%)	648 (77.3%)	5.8 (4.9–6.9)	<0.0001	5.0 (4.1–6.0)	<0.0001
	≥37+0	1.709 (20.4%)	6.689 (79.6%)	4.1 (3.9–4.4)	<0.0001	3.6 (3.4–3.8)	<0.0001
**Brachial plexus injury**	24+0 to 27+6*	0 (0.0%)	0 (0.0%)	-	-	-	-
	28+0 to 31+6	1 (100.0%)	0 (0.0%)	-	-	-	-
	32+0 to 36+6	2 (66.7%)	1 (33.3%)	0.7 (0.08–3.2)	0.770	0.7 (0.05–7.9)	0.750
	≥37+0	10 (23.8%)	32 (76.2%)	2.8 (1.4–5.7)	0.004***	4.1 (1.9–9.02)	0.0004
**Eye injury**	24+0 to 27+6*	0 (0.0%)	0 (0.0%)	-	-	-	-
	28+0 to 31+6	0 (0.0%)	0 (0.0%)	-	-	-	-
	32+0 to 36+6	1 (20.0%)	4 (80.0%)	5.0 (1.2–8.2)	0.120	5.2 (0.5–48.6)	0.14
	≥37+0	29 (6.4%)	426 (93.6%)	13.1 (9.0–19.1)	<0.0001	13.3 (9.06–19.7)	<0.0001
**Facial injury**	24+0 to 27+6*	0 (0.0%)	0 (0.0%)	-	-	-	-
	28+0 to 31+6	0 (0.0%)	1 (100.0%)	-	-	-	-
	32+0 to 36+6	3 (50.0%)	3 (50.0%)	1.4 (0.8–1.5)	0.682	1.4 (0.2–10.8)	0.736
	≥37+0	2 (66.7%)	1 (33.3%)	0.4 (0.02–4.2)	0.504	0.4 (0.03–4.5)	0.443
**Fetal laceration by scalpel**	24+0 to 27+6*	0 (0.0%)	0 (0.0%)	-	-	-	-
	28+0 to 31+6	0 (0.0%)	0 (0.0%)	-	-	-	-
	32+0 to 36+6	12 (100.0%)	0 (0.0%)	-	-	-	-
	≥37+0	213 (97.3%)	6 (2.7%)	0.02 (0.006–0.1)	<0.0001	0.07 (0.02–0.14)	<0.0001
**Meconium and/or amniotic fluid aspiration**	24+0 to 27+6*	0 (0.0%)	0 (0.0%)	-	-	-	-
	28+0 to 31+6	0 (0.0%)	0 (0.0%)	-	-	-	-
	32+0 to 36+6	5 (62.5%)	3 (37.5%)	0.8 (0.2–3.5)	0.81	0.9 (0.1–6.2)	0.930
	≥37+0	122 (29.4%)	293 (70.6%)	2.1 (1.7–2.6)	<0.0001	1.8 (1.4–2.2)	<0.0001
**Subcutaneous fat necrosis of the newborn**	24+0 to 27+6*	0 (0.0%)	0 (0.0%)	-	-	-	-
	28+0 to 31+6	0 (0.0%)	0 (0.0%)	-	-	-	-
	32+0 to 36+6	5 (100%)	0 (0.0%)	-	-	-	-
	≥37+0	56 (81.2%)	13 (18.8%)	0.2 (0.1–0.4)	<0.0001	0.2 (0.1–0.4)	<0.0001
**Ecchymosis**	≤36+6	42 (0.9%)	115 (3.1%)	3.6 (2.5–5.1)	<0.0001	3.6 (2.4–5.4)	<0.0001
	>37+0	591 (2.1%)	2.917 (9.4%)	4.7 (4.3–5.1)	<0.0001	4.7 (4.3–5.2)	<0.0001
**Birth injury to spleen**	≤36+6	0 (0.0%)	0 (0.0%)	-	-	-	-
	>37+0	2 (100%)	0 (0.0%)	-	-	-	-

Abbreviations: OR, unadjusted odds ratio, aOR adjusted odds ratio for maternal age, parity, ethnicity, gestational age at delivery, and maternal comorbidities.

“Other scalp injury” birth trauma category includes other intracranial lacerations and haemorrhages: petechiae, excoriations, scalp ecchymoses, cephalhematoma, caput succedaneum

^1^ Data presented as n (%).

*Reference category

**p<0.001

***p<0.05.

^a^Sample size does not equal to 62.330 due to missing data

There were no significant differences in the rates of cranial traumatic haemorrhage (cerebral, subarachnoid, tentorial, intracranial and the ventricular haemorrhage) in term and preterm neonates between CS and VD. The “*other scalp injury*” birth trauma category, that included all minor injuries, accounted for the majority of all birth trauma (75% of all birth trauma). When comparing CS to VD, and term to preterm deliveries, odd ratios of all birth scalp traumas were higher for VD (aOR 4.1, 95% CI 3.9–4.4, p<0.0001) and term deliveries (aOR 3.6, 95% CI 3.4–3.8, p < .0001).

The occurrence rates of intraventricular haemorrhage were similar among VD and CS groups, when stratified by gestational age of delivery groups. Only one case of cranial nerve lesion was identified, and this was a term newborn. Regarding fractures at birth, the majority of clavicle fractures were recorded for term births (OR 14.9, 95% CI 9.6–23.2, p <0.0001), fewer cases for the 32–36 weeks group, and only one case for very low preterm. For moderate to late preterm group, after adjusting for maternal clinical characteristics, VD continued to be associated with increased odds (9.2 vs 4.2) of fracture of clavicle. Brachial plexus injury was more frequent in term births (42 vs 4 cases) compared to preterm births, and for VD compared to CS (aOR 4.1, 95% CI 1.9 1.8–9.0, p = 0.004).

Eye injury due to trauma at birth (chemosis, subconjunctival haemorrhage, localized bruising) were more frequent for term neonates delivered vaginally (93.6% in VD; aOR 13.1, 95% CI 9.1–19.7, p <0.0001). There was no difference in fetal facial laceration in VD and CS for all delivery age groups.

Meconium aspiration syndrome was reported in late preterm and term deliveries. Among term neonates, there was a 70.6% to 29.4% (aOR 1.8, 95% CI 1.4–2.2, p<0.0001) difference between VD and CS. Subcutaneous fat necrosis of the newborn was more common after CS, in full-term and late preterm births. Ecchymoses were more common in term compared to preterm deliveries (96.0% vs 4.0%, p <0.0001), and after VD compared to CS (aOR 4.7, 95% CI 4.3–5.2, p<0.0001).

## Discussion

The aim of the current study was two-fold, to assess the incidence and characteristics of birth trauma in units from our country, and to contribute to the existing literature on perinatal trauma, thus strengthening the quality of evidence in support of clinical protocols and guidelines worldwide regarding the safest mode of delivery in preterm pregnancies, with fetuses in vertex presentation. In spite of recent research accruing data on the optimal mode of delivery for preterm and very preterm infants, there is still not enough information to provide clear guidance on the chosen method of birth in preterm pregnancies and a consensus has not been yet achieved [5].

Because a RCT was not proven to be feasible, the evidence on birth trauma is based mostly on retrospective studies. Therefore, we conducted a retrospective study on a large population of 62,323 women that compared neonatal birth injuries in term and preterm births, in vaginal versus caesarean delivery. The most important findings of our study are as follow: i) the incidence of birth trauma was 16.2 per 1000 births; ii) there is an increasing trend of delivery of preterm babies by CS (from 37.0% of total preterm births in 2007 to a percentage of 60.0% in 2017); iii) the incidence of trauma at birth is higher after 28 weeks gestation, in VD compared to delivery by CS; iv) preterm babies are not a higher risk of major birth trauma if they are delivered vaginally compared to delivery by CS; v) there was no difference in the incidence of birth trauma before 28 weeks gestation in babies delivered vaginally or by CS.

When we stratified by type of birth trauma we observed that: i) there was no significant difference for minor trauma between preterm and term infants delivered either by CS or VD; ii) fetal birth hypoxia was more common in term babies delivered by CS, whereas asphyxia was more common in preterm birth delivered by CS; iii) low Apgar scores were more common in late preterm delivered by CS and term babies delivered vaginally; iv) there was difference in duration of hospitalization of mothers between CS and VD, but not in the duration of hospitalization of the newborns.

The total rate of CS in our study was 45.2%, which is a high incidence compared to other European countries (i.e.22% in Belgium) [11]. Recent analysis found an increase trend of caesarean deliveries in all regions except sub-Saharan Africa, with the global average increasing from 6.7% in 1990 to 19.1% in 2014. In Europe, the caesarean section rates have risen to 24% - 30% today and are projected to continue increasing over this current decade. Conversely, in Latin America and the Caribbean, rates are as high as 4 in 10 (43%) of all births. In five countries (Dominican Republic, Brazil, Cyprus, Egypt and Turkey), caesarean sections outnumber vaginal deliveries [12].

Although, CS is safer today and it is now commonly performed to protect the fetus, newborn morbidity and mortality still do exist [13]. A cohort study of singleton, live born, in cephalic presentation deliveries, between 24 and 34 weeks gestation, CS delivery was not protective against poor outcomes and in fact was associated with increased risk of respiratory distress and low Apgar score compared to VD [14]. Increase in the rate of CS was associated with a higher number of babies admitted to intensive care for 7 days or longer even after adjustment for preterm delivery. Rates of preterm delivery and neonatal mortality both rose at rates of CS between 10% and 20%, suggesting that CS do not necessarily indicate better perinatal outcomes and can be associated with neonatal trauma [15].

There are several factors that may help explaining the high rates of delivery by CS among present cohort of preterm and term neonates. Along with the fear of malpractice, lesser experience of young obstetricians in instrumental deliveries, increased usage of epidural analgesia during labor, an increased desire of women for CS delivery is noted among our obstetrical population. It is also important to mention that the center where the study was conducted is a level 3 maternity hospital and a referral center for higher risks pregnancies and women with comorbidities. In addition, in Romania, the induction of labour with prostaglandins is not part of labour management guidelines, thus leading to higher incidence of caesarean births.

In our cohort, the rate of preterm birth was 10.9% and that of birth trauma 16.2%. Our reporting included all types and degrees of severity of birth trauma and injuries. The rate of birth trauma reported by others was about 2% in normal VD in cephalic presentation and 1.1% in CS, but included only selective reporting of various types of birth trauma [6, 16, 17].

Some studies have shown lower risk of birth trauma for CS delivery [1, 18, 19]. Charmaine et al. showed a percentage of all birth trauma of 25.85 per 1000 births in a population of over 8 million births in US [2]. Furthermore, other authors pointed out that the incidence of common birth trauma injury was 41.16 per 1000 vaginal deliveries of singleton fetuses in vertex presentation [3]. Also, these authors found that rates of birth injury ranged from 0.3–3.8 per 1000 live births and were distributed by birth types as follow: 0.3 of 1000 CS; 1 of 1000 VD, and 3 of 1000 operative and instrumented VDs [3]. Manuck et al. found in a study cohort of 8334 deliveries an incidence of 1.4% neonatal deaths, 7.9% major morbidity, 63.1% survival without any morbidity, and 37.6% minor morbidity [20].

Compared to other studies, we found a higher rate of overall birth injuries. If we were to exclude the most common minor injuries that represented 75% of all injuries, the calculated incidence rate of birth trauma would have become 40.55 per 1000 births (2,528 cases with various degrees of significant traumas from a cohort of 62,330 births), index similar with that reported by other studies. Major traumas were rare and no statistically significant differences was seen between VD and CS. This is an agreement with the findings reported by a Cochrane systematic review on the outcomes of birth in preterm infants which reported a risk ratio of 0.56, with a 95% CI of 0.05 to 5.62 for infant birth injury when comparing planned immediate CS and planned VD in preterm births [5].

We report no difference in our study in the incidence of intracranial lacerations and cranial traumatic haemorrhage (cerebral, subarachnoid, tentorial, intracranial, and ventricular haemorrhage) in preterm pregnancies delivered either by CS or VD at any gestational age. Previous studies have produced conflicting results, with some suggesting a protective effect of CS delivery, [7, 9, 21] whereas others found a reduction in mortality and intraventricular hemorrhage for the infants born before 34 weeks via VD [8]. Intraventricular hemorrhages, especially in premature infants, are not considered traumatic [22]. However, we took them into account because there were studies that showed differences in preterm groups <32 weeks between CS and VD birth, suggesting that CS is a protective factor, and therefore that there is a traumatic component that would influence the results. However, we did not find significant differences between CS and VD in preterm births <32 weeks.

Nerve injuries were rare, with brachial plexus injury being most common. In this study we observed an increase in this type on injury with gestational age, with the majority of these type of injury occurring after 36 weeks gestation, and almost three times more frequent in VD delivery. Others report similar findings, with CS being protective of nerve injury [23].

### Strengths and limitations

The main limitation of our study is the retrospective design. The data was retrieved from electronic and handwritten patient records. The data is stored in the electronic records based on administrative criteria, which inevitably led to unavailability of some desirable information or misclassification. There was no data available on the induction of labour or detailed circumstances leading to decision on CS delivery. Furthermore, we used the most recent ICD classification, ICD 10, although it is possible that misclassification may have occurred due to use previous ICD codes in years preceding our study.

Not reporting on mortality rates was another limitation of our study. We did report, however, on the rate of stillbirth, that was 0.67% in our study, comparable to previous reports [24, 25].

This study has several strengths. We have evaluated the impact of VD and CS delivery in a large sample of term and preterm pregnancies. Additionally, the database used combined maternal and neonatal information, allowing us to ensure the accuracy of data collected. Furthermore, the importance of this study is two-fold. Firstly, to date, there are no other studies conducted in Romania to assess the rate and consequences of birth trauma at national level. This study conducted in one the largest tertiary maternity center in the country is representative and can be generalised to the entire population of the country. This is more significant if considered that Romania is the country with highest incidence of premature births, of about 10–12%, and one of the highest incidences of CS (from 34% in 2014 to exceeding 60% in 2018) in Europe. Thus, it is important to understand the causes driving this increase, being these supported by clinical evidence or driven solely by fear of mass media and medical error with consequent reprimand and liability. Secondly, our analysis of mode of delivery aligns with the trend observed worldwide in assistance at delivery, with a dramatic increase in the rates of CS and decrease in instrumental deliveries, driven mostly by socio-economic factors. The rate of instrumental delivery, less than 1% in our centre, allowed us to exclude these women from the analyses, while enabling comparison of VD and CS and estimation of birth trauma without the bias of trauma by forceps and vacuum. Thirdly, vacuum assisted vaginal delivery is contraindicated in pregnancies less than 34 weeks gestational age or estimated fetal weight lower than 2500 gm.

## Conclusions

In summary, our study does not support benefits of CS over VD for premature newborns in order to avoid major birth trauma. Therefore, we advise the decision on the method of delivery of preterm babies based on obstetric indications, while considering the benefits and urgency of fetal delivery. Although the incidence of total birth trauma favor CS delivery, severe birth trauma develops equally frequent in CS and VD for preterm babies in vertex presentation. This led to the conclusion that overall, the evidence to date remains controversial and, therefore, prospective studies are needed to explore whether CS in preterm births protects against birth trauma. Pending future studies, these findings suggest that CS does not further protect the singleton premature newborn in cephalic presentation from major trauma and that VD can be considered.

## Supporting information

S1 Table ICD-10 codes for neonatal outcomes. (DOCX)

S2 Table ICD-10 codes for maternal comorbidities. (DOCX)
