# Supplementary material for: Birth trauma in preterm spontaneous vaginal and cesarean section deliveries: A 10-years retrospective study
Source: PLoS One. 2022 Oct 17;17(10):e0275726. doi: 10.1371/journal.pone.0275726 (PMC9576096; doi:10.1371/journal.pone.0275726)
Supplement: S1 Table — (DOCX) [file pone.0275726.s001.docx]

**S1 Table. ICD-10 codes for neonatal outcomes**

| **ICD- 10 CODES** | **Neonatal birth outcomes** |
| --- | --- |
| P10 | Intracranial laceration and haemorrhage due to birth injury |
| P10.0 | Subdural haemorrhage due to birth injury |
| P10.1 | Cerebral haemorrhage due to birth injury |
| P10.2 | Intraventricularhaemorrhage due to birth injury |
| P10.3 | Subarachnoid haemorrhage due to birth injury |
| P10.4 | Tentorial tear due to birth injury |
| P10.8 | Other intracranial lacerations and haemorrhages due to birth injury |
| P10.9 | Unspecified intracranial laceration and haemorrhage due to birth injury |
| P11 | Other birth injuries to central nervous system (including: cerebral oedema, cranial nerves, spine or spinal cord) |
| P11.3 | Birth injury to facial nerve |
| P12 | Birth injury to scalp |
| P12.0 | Cephalhaematoma due to birth injury |
| P12.1 | Chignon due to birth injury |
| P12.2 | Epicranialsubaponeurotichaemorrhage due to birth injury |
| P12.3 | Bruising of scalp due to birth injury |
| P12.4 | Monitoring injury of scalp of newborn (Sampling incision, Scalp clip (electrode) injury) |
| P13 | Birth injury to skeleton |
| P13.2 | Birth injury to femur |
| P13.3 | Birth injury to other long bones |
| P13.4 | Fracture of clavicle due to birth injury |
| P14.3 | Other brachial plexus birth injuries |
| P15.0 | Birth injury to liver |
| P15.1 | Birth injury to spleen |
| P.15.3 | Birth injury to eye (Subconjunctivalhaemorrhage and Traumatic glaucoma due to birth injury) |
| P15.4 | Birth injury to face (Facial congestion due to birth injury) |
| P15.6 | Subcutaneous fat necrosis due to birth injury |
| P15.9 | Other specified birth injuries (ex. fetal laceration by scalpel) |
| P24.1 | Neonatal aspiration of amniotic fluid and mucus |
| P24.2 | Neonatal aspiration of blood |
| P20 | Intrauterine hypoxia (abnormal fetal heart rate; fetal or intrauterine: acidosis, anoxia, asphyxia, distress, hypoxia; meconium in liquor) |
| P20.9 | Intrauterine hypoxia, unspecified |
| P21 | Birth asphyxia (*Note:* This category is not to be used for low Apgar score without mention of asphyxia or other respiratory problems) |
| P21.0 | Severe birth asphyxia (Pulse less than 100 per minute at birth and falling or steady, respiration absent or gasping, colour poor, tone absent; Asphyxia with 1-minute Apgar score 0-3; White asphyxia) |
| P21.1 | Mild and moderate birth asphyxia (Normal respiration not established within one minute, but heart rate 100 or above, some muscle tone present, some response to stimulation; Asphyxia with 1-minute Apgar score 4-7; Blue asphyxia) |
| P21.9 | Birth asphyxia, unspecified (anoxia, asphyxia, hypoxia ) |
| P54.5 | Neonatal cutaneous haemorrhage (bruising, ecchymoses, petechiae, superficial haematoma) |
