# Supplementary material for: Birth trauma in preterm spontaneous vaginal and cesarean section deliveries: A 10-years retrospective study
Source: PLoS One. 2022 Oct 17;17(10):e0275726. doi: 10.1371/journal.pone.0275726 (PMC9576096; doi:10.1371/journal.pone.0275726)
Supplement: S2 Table — (DOCX) [file pone.0275726.s002.docx]

**S2 Table. ICD-10 codes for maternal comorbidities**

| **ICD-10 codes** | **Patology** |
| --- | --- |
| O24 | Diabetes mellitus in pregnancy |
| O24.0 | Pre-existing type 1 diabetesmellitus |
| O24.1 | Pre-existing type 2 diabetesmellitus |
| O24.4 | Diabetes mellitus arising in pregnancy |
| O10 | Pre-existing hypertension complicating pregnancy, childbirth and the puerperium |
| O11 | Pre-eclampsia superimposed on chronic hypertension |
| O13 | Gestational [pregnancy-induced] hypertension |
| O14 | Pre-eclampsia |
| O15 | Eclampsia |
| O42 | Premature rupture of membranes |
| O41.1 | Infection of amniotic sac and membranes |
| O23.5 | Infections of the genital tract in pregnancy |
